# Role of COVID-19 Anxiety and Community Risk Factors on Physical Distancing Practice

**DOI:** 10.3390/bs12040110

**Published:** 2022-04-16

**Authors:** Hsin-Yi Wang, Cecilia Cheng

**Affiliations:** Social and Health Psychology Laboratory, Department of Psychology, The University of Hong Kong, Pokfulam, Hong Kong, China; hylwang1688@gmail.com

**Keywords:** anxiety, coronavirus, epidemic, novel disease, pandemic, preventive measures

## Abstract

Existing studies have focused primarily on self-oriented anxiety (i.e., anxiety over one’s infection) in the pandemic context, and the role of community risk is largely ignored. This study addressed these gaps by examining (a) self-oriented anxiety and two forms of others-oriented anxiety (i.e., anxiety concerning others’ health and societal problems), (b) the associations between all these forms of anxiety and physical distancing practice during the COVID-19 pandemic, and (c) the hypothesized moderating role of community risk factors. The participants were 703 U.S. community-dwelling adults who completed an online survey. Geo-identifier data were extracted to identify the number of confirmed COVID-19 cases and four social vulnerability indexes for the county in which each participant resided. Both forms of others-oriented anxiety were positively associated with physical distancing adoption, and the association was stronger among the participants residing in lower-risk communities (i.e., fewer confirmed COVID-19 cases, higher socioeconomic status, and better housing conditions). The study’s novel findings reveal the protective role of anxiety, particularly anxiety concerning others’ well-being, in encouraging people to adopt physical distancing during a pandemic. However, the protective role of anxiety is contingent upon certain community risk factors. Anxiety is more beneficial to residents of low- rather than high-risk communities.

## 1. Introduction

The COVID-19 pandemic presents an unprecedented challenge to societies all over the globe [1,2], and changes in daily social habits are necessary to curb the further spread of this highly contagious disease. Given the possibility of asymptomatic transmission, many governments and public health institutions have recommended the practice of physical distancing, or reducing close contact with others, to prevent COVID-19 infection [3,4]. The primary goal of this preventive measure is to attenuate the likelihood of individuals who have been infected spreading the disease in the community, thereby reducing public demand for healthcare services.

Despite the escalating number of confirmed COVID-19 cases worldwide, many individuals remain unwilling to practice physical distancing on a voluntary basis. A possible explanation is that, relative to other preventive measures (e.g., wearing facemasks, washing hands), physical distancing comes at a much higher personal cost, as it requires people to drastically alter their lifestyles and avoid face-to-face contact with others [5]. However, such non-compliant behavior can hinder collective efforts to curb disease transmission. To elicit greater public support for the routine practice of physical distancing, the psychological factors associated with its take-up need to be identified. Studying such factors will aid the design of disease control interventions while the COVID-19 pandemic is still underway.

### 1.1. COVID-19 Anxiety and Physical Distancing Practice

Anxiety has been documented as a major psychological factor influencing the tendency to deploy strategies for preventing a disease infection [6,7]. For instance, stress appraisal theory postulates that individuals with high levels of anxiety are prone to perceiving potential stressors as threatening [8]. In line with that theory, anxiety over the H1N1 pandemic was found to be positively associated with strongly negative appraisals of the pandemic threat [9]. Other studies of that earlier pandemic demonstrated that people who perceived stressors as threatening were more motivated to engage in precautionary behaviors to protect themselves than those who were less concerned [10,11]. High levels of pandemic-specific anxiety may, thus, elicit perceptions of the pandemic as dangerous, thereby strengthening the motivation to undertake preventive measures, such as physical distancing to avoid infection.

A study conducted during the COVID-19 pandemic showed that anxiety over potential infection was associated with a greater propensity to engage in physical distancing behavior and comply with government-mandated restrictions [12]. Moreover, anxiety-provoking messages emphasizing the adverse outcomes of the pandemic (e.g., severe symptoms, a high death toll) have been found to increase people’s willingness to self-quarantine [13]. However, many of these studies espouse an individual approach that focuses on self-oriented anxiety, pertaining to feeling personally at risk of infection and the corresponding symptoms of such anxiety [14,15].

The present study seeks to broaden the scope of the extant literature in this area by investigating anxiety over the wellbeing of one’s social network members and society as a whole. As the viral transmission and the infection rates were both unusually high for COVID-19 [16], people from COVID-19-affected regions expressed concerns not only about themselves, but also others in their social network and community [17,18]. For instance, facemask wearing in public areas is found to be predicted by motives pertaining to not only oneself (i.e., fear of one’s infection, perceived comfort when wearing facemasks), but also others (i.e., fear of infecting others, social norms) [19]. Moreover, despite having considerable hesitancy in the newly developed COVID-19 vaccines, regarding uncertain efficacies and side effects, many people expressed willingness to vaccinate due to their worries about infecting people living in the same household or in their community [20,21]. The investigation of others-oriented anxiety is essential in studying physical distancing adoption because such a preventive measure attenuates the risk of disease contraction, not only for the individuals adopting that measure, but also for others [22].

In a study that adopted a nuanced approach for examining pandemic-related anxiety, residents from COVID-19-hard-hit countries were asked to report what made them anxious since the onset of the pandemic [23]. Apart from concerns over one’s own wellbeing, most of the residents also reported concerns over the wellbeing of members of their social network, including whether members of their social network and those in their community would contract COVID-19. Besides, many also reported concerns about various societal issues, such as possible breakdown of the local healthcare system, economic aftermath of the pandemic, and the wealth–health gap. These two alternative dimensions of anxiety were found to be positively associated with perceived likelihood and impact of a COVID-19 infection, and also with another major mental health indicator of depression [24]. Accordingly, this nuanced approach to study of the association between these alternative dimensions of anxiety and the frequency of physical distancing adoption was applied in this research. Anxiety concerning both others’ health and societal problems was hypothesized to be positively associated with such frequency, beyond the influence of self-oriented anxiety about one’s own potential infection.

### 1.2. COVID-19 Anxiety and Community Risk Factors

Although some people are more predisposed than others to feel anxious when encountering a health crisis, the experience of pandemic-related anxiety and the implications thereof can also be influenced by environmental factors [21,25]. This notion stems from previous disaster research, which has revealed that sharing distress among community members experiencing a natural disaster tends to increase prosocial responses, as such experiences strengthen one’s social identification with the community and empathetic concern toward other in-group members [25]. Similar evidence obtained amidst the COVID-19 pandemic has indicated considerable variations in residents’ motivation to vaccinate across regions with diverse social density [21]. Specifically, people living in regions with lower social density tend to show greater acceptance and willingness to receive a COVID-19 vaccine due to their stronger beliefs that their adoption of this prevention measure would have a greater impact on members of their society. In light of these findings, environmental factors are particularly likely to play an influential role in the current pandemic because communities have been exposed to differing levels of health risk, particularly during the pandemic’s early stages. Hence, knowledge of the pandemic-related thoughts and behaviors of people living in diverse communities across the United States, particularly those of the residents of high-risk communities, is pivotal to slowing the spread of COVID-19. The acquisition of such knowledge may foster the more effective promotion of preventive measure adoption among the general public.

To address the aforementioned unexplored issues, two broad community risk factors were examined: confirmed COVID-19 cases and social vulnerability. Within the United States, the number of confirmed COVID-19 cases varies dramatically from state to state. At the initial wave of the pandemic, New York state was the hardest hit, with over 390,000 confirmed cases, followed by California, with more than 191,000 cases, whereas other states, including Alaska and Montana, have fewer than 1000 cases each [26]. The residents of communities with a higher number of confirmed COVID-19 cases are at a much greater risk of coming into contact with people who have contracted the disease than their counterparts in communities with fewer cases.

U.S. communities also differ considerably with respect to social vulnerability, which refers to the social conditions affecting a community’s capacity to prevent human suffering in the event of a disaster (e.g., a pandemic). To quantify such vulnerability, the U.S. Centers for Disease Control and Prevention created the Social Vulnerability Index (SVI), a percentile-based index of county-level vulnerability across the United States [27]. The SVI comprises four underlying themes. Theme 1 (socioeconomic status (SES)) measures such factors as the poverty rate and unemployment rate. Theme 2 (household composition) evaluates the proportion of the elderly, children, individuals with disability, and single-parent households in a community. Theme 3 (ethic minority status) assesses the proportion of ethnic minorities in a community and the corresponding language barriers. Finally, Theme 4 (housing type) evaluates factors related to poor housing conditions in a community, including a large percentage of mobile homes, multi-unit structures, and over-crowded housing. Such vulnerabilities, including a low SES, lack of workplace protections, language barriers, and household overcrowding have been associated with poor healthcare access and poor adherence to government-instructed health guidelines [28,29].

### 1.3. A Person–Environment Interactionist Approach

The study reported herein adopted a person–environment interactionist approach to investigate the interplay of others-oriented anxiety and community risk factors in physical distancing adoption. The major tenet of the person–environment interactionist approach is that differences among people with personality characteristics are moderated by certain environmental factors, and the conjoint effects of both personality and environmental factors exert the strongest effect on individuals’ wellbeing [30,31]. In the literature, the person–environment interactionist model has been widely adopted in anxiety research, which reveals an interplay of certain environmental variants and individuals’ dispositional tendency on the experience of anxiety symptoms, in an array of stressful contexts, such as social separation, tests and examinations, sports competition, and information technology use [32,33,34,35]. Applying this approach to the present pandemic context, we propose that the association between both forms of others-oriented anxiety, namely, anxiety pertaining to others’ health and anxiety over societal problems, and the frequency of physical distancing adoption, to be stronger among the residents of low-risk communities (i.e., those with relatively few confirmed COVID-19 cases and a low SVI score) than among the residents of high-risk communities. This hypothesis was formulated on the basis of evidence suggesting that social vulnerabilities, such as chronic economic deprivation, can heighten negative attitudes among those living in the affected communities, thereby reducing levels of self-efficacy and perceived control over life events [36,37]. A similar phenomenon has also been observed among those living in regions heavily affected by the COVID-19 pandemic. For instance, a recent study conducted in China during the early stage of the COVID-19 pandemic found individuals residing in the epicenter province of Hubei to report lower levels of both self-efficacy and preventive measure adoption than residents of less-affected provinces [38]. These studies suggest that high levels of COVID-19 anxiety among the residents of high-risk communities may not necessarily increase their engagement in physical distancing behaviors because of their negative attitudes, which constitute an important barrier to the intention to adopt health-related preventive measures [39,40].

On the basis of previous theories and studies, the following hypotheses were proposed and tested in this study:

**Hypothesis** **1** **(H1).** *Others-oriented anxiety (i.e., anxiety concerning others’ health and anxiety concerning societal problems) would be positively associated with physical distancing adoption.*

**Hypothesis** **2** **(H2).** *The association between others-oriented anxiety and physical distancing adoption would be moderated by the number of confirmed COVID-19 cases, such that the association would be stronger (vs. weaker) among individuals living in counties with a low (vs. high) number of confirmed cases.*

**Hypothesis** **3** **(H3).** *The association between others-oriented anxiety and physical distancing adoption would be moderated by social vulnerabilities, such that the association would be stronger (vs. weaker) among individuals living in counties with more (vs. fewer) such vulnerabilities.*

## 2. Materials and Methods

### 2.1. Sample and Procedures

Participants were recruited from Prolific Academic, an online research platform that recruits participants with diverse demographic characteristics. Adults aged 18 to 65 who were residing in the United States were eligible to participate in the study. To ensure high-quality data, only those who had had at least 95% of their work approved by the platform could take part. The participants were recruited to approximate the sex and age distribution of the U.S. population and were required to provide informed consent prior to study commencement. The compensation scheme (USD 1 for 10 min) adhered to Prolific Academic’s regulations. A total of 711 participants completed the survey. On the basis of geo-identifier data recorded by the online platform, eight participants were found to be outside U.S. territory at the time of the study, and their data were thus excluded.

Data collection was conducted from 18–19 March 2020, via an online survey hosted by Qualtrics. The study protocol received prior ethical approval from the institutional review board of the authors’ university.

### 2.2. Measures

#### 2.2.1. Others-Oriented Anxiety

On the basis of our previous work [23], items were constructed to measure two sources of others-oriented anxiety experienced during the COVID-19 pandemic, with five items measuring anxiety over others’ health (e.g., COVID-19 infection among my family members, health problems of elderly people in my community) and three measuring anxiety over societal problems (e.g., breakdown of the healthcare system, widening of society’s health–wealth gap). This new measure has been found to display adequate internal consistency and concurrent validity [24]. To further examine the structural validity of this two-factor measure, confirmatory factor analysis was performed in R version 3.6.0 with lavaan version 5.20. The results revealed good structural validity for the present sample (CFI = 0.971, TLI = 0.958, RMSEA = 0.059, SRMR = 0.068), as well as good and acceptable internal consistency for the two subscales (Cronbach’s alpha = 0.841 for anxiety concerning other’s health and 0.763 for anxiety concerning societal problems).

#### 2.2.2. Self-Oriented Anxiety

A three-item measure was used to assess self-oriented anxiety concerning COVID-19 infection (e.g., How nervous do you feel about contracting COVID-19?). These items were adapted from a measure developed and validated during the outbreak of severe acute respiratory syndrome (SARS) [41]. Participants were instructed to rate the three items on a 4-point scale (1 = not at all; 4 = very). The measure displayed acceptable internal consistency in the present study (Cronbach’s alpha = 0.744).

#### 2.2.3. Community Risk Factors

Five community risk factors were examined at the county level: number of confirmed COVID-19 cases and four SVI scores (i.e., SES, household composition, ethnic minority status, and housing type). The SVI scores were matched with the participants’ current locations (counties), which were identified using Google’s reverse geocoding procedure to translate the geo-identifier data (i.e., longitude and latitude) into readable addresses [42].

Data on the number of confirmed COVID-19 cases were obtained from a database maintained by John Hopkins Coronavirus Resource Center [26], with daily figures extracted for each county from 11–17 March 2020. The daily figures were then averaged across the week. As the data for this study were collected during an early stage of the COVID-19 pandemic, there were substantial variations in the number of confirmed COVID-19 cases across states and counties, with far greater numbers in major metropolitan areas such as New York City. To reduce the potential bias produced by outliers, rank transformation was applied to the number of COVID-19 cases. Thirty-four unique ranked values were identified, ranging from 0 (Rank 1) to 342 (Rank 34) cases. The four SVI scores were extracted from the dataset maintained by the U.S. Centers for Disease Control and Prevention [27].

#### 2.2.4. Physical Distancing Adoption

The frequency of physical distancing adoption was measured using an instrument developed and validated during the SARS outbreak [27]. Physical distancing was indicated by four avoidance behaviors: avoiding dining out, avoiding going out to shop, avoiding shaking hands, and avoiding contact with people displaying COVID-19 symptoms. Participants rated the frequency with which they had adopted such behaviors in the past week on a 4-point scale (0 = never; 1 = 1–2 times; 2 = 3–4 times; 3 = 5 or more times). The scale was found to be reliable in this study (Cronbach’s alpha = 0.781).

#### 2.2.5. Demographic Characteristics

Participants were asked to provide information on their age, sex, education level (tertiary education or above vs. high school diploma or below), annual income (in USD) (<20,000, 20,001–60,000, 60,001–100,000, and >100,001), and employment status (full-time employee, part-time employee, and currently not working).

### 2.3. Data Analysis

Demographic differences in the frequency of physical distancing adoption were examined using the following statistical methods. Pearson’s correlation analysis was conducted for age differences, and independent sample *t*-tests for differences in sex and educational level. Finally, one-way analysis of variance was performed to analyze differences pertaining to income level and employment status.

Hierarchical multiple regression analysis was conducted to test all of the hypotheses, including the hypothesized direct associations between others-oriented anxiety and physical distancing adoption, as well as the hypothesized interaction between the others-oriented anxiety and community risk variables on the criterion variable of physical distancing adoption. Two sets of regression analysis were conducted to examine the two forms of others-oriented anxiety (concerning others’ health and societal problems) separately. In the first step, self-oriented anxiety and the demographic variables identified as significantly associated with physical distancing adoption were entered as covariates. In the second step, each form of others-oriented anxiety and the five community risk variables were entered to test Hypothesis 1 (H1). In the third step, the five interaction terms (each with a multiplication term of one form of anxiety and one community risk variable) were entered to test the hypothesized moderating role of the various community risk variables (Hypothesis 2 (H2) and Hypothesis 3 (H3)).

If a significant interaction effect was found, the estimate of the regression slope at one standard deviation above and below the centered mean of the moderators was computed. This analysis was conducted separately for each significant interaction effect. In addition, a test of the simple slopes was carried out by computing their standard errors. The aforementioned analyses were conducted in R version 3.6.0 [43].

## 3. Results

### 3.1. Sample Characteristics

The final sample comprised 703 participants, 50.9% of whom were women. The mean age was 42.2 years (SD = 13.3, age range = 18–65). Approximately half (50.5%) of the participants had received tertiary education, and a similar proportion (49.1%) were full-time employees. The median range of annual household income was USD 30,001–40,000.

With respect to the number of confirmed COVID-19 cases, 29.4% of the participants were residing in counties with fewer than five confirmed cases at the time of the study, whereas 31.0% and 40.3% were living in counties with 6–50 and 50+ confirmed cases, respectively. The following proportions of participants were also residents of highly vulnerable communities (counties scoring in the top 10% nationwide): 23.7% for SVI Theme 1 (SES), 34.2% for SVI Theme 2 (household composition), 32.1% for SVI Theme 3 (ethnic minority status), and 41.6% for SVI Theme 4 (housing type).

### 3.2. Demographic Differences in Physical Distancing Adoption

The only demographic variable found to be positively associated with physical distancing adoption was age (*r* = 0.136, *p* < 0.001), with older (younger) participants reporting that they tended to practice physical distancing more (vs. less) frequently (all of the other demographic variables had *p*s > 0.05).

### 3.3. Association of Others-Oriented Anxiety and Community Risk Variables with Physical Distancing Adoption

The results of hierarchical regression analysis are summarized in Table 1. Both anxiety over others’ health and anxiety over societal problems were positively associated with physical distancing adoption, after controlling for self-oriented anxiety about one’s own potential COVID-19 infection, thereby offering support for Hypothesis 1 (H1). The positive association between anxiety over societal problems and physical distancing adoption was moderated by the number of confirmed COVID-19 cases, which provides partial support for Hypothesis 2 (H2), because this moderating effect was not found to be significant for anxiety over other’s health. In addition, the positive association between both forms of others-oriented anxiety and physical distancing adoption was moderated by two of the SVI themes, namely, SES and housing type, providing partial support for Hypothesis 3 (H3).

Simple slope analysis conducted to probe these interactions revealed a significantly positive association between anxiety over others’ health and physical distancing adoption among residents of counties with a higher SES (*B* = 0.344, *SE* = 0.039, *p* < 0.001), but the association was not significant for those living in counties with a lower SES (*B* = 0.104, *SE* = 0.032, *p* = 0.272). In addition, there was a significant association in counties with better housing conditions (*B* = 0.313, *SE* = 0.039, *p* = 0.004). A similar, albeit weaker, positive correlation was found in those with worse housing conditions (*B* = 0.121, *SE* = 0.033, *p* = 0.032).

The findings were similar for the association between anxiety over societal problems and physical distancing adoption. Simple slope analysis revealed a significantly positive association in counties with a higher SES (*B* = 0.378, *SE* = 0.041, *p* < 0.001), and a weaker positive association was identified among those residing in communities with a lower SES (*B* = 0.120, *SE* = 0.032, *p* = 0.028). A positive association was also found among residents of counties with better housing conditions (*B* = 0.400, *SE* = 0.043, *p* < 0.001). The association was not significant for those living in communities with worse housing conditions (*B* = 0.082, *SE* = 0.025, *p* = 0.34), however. Moreover, for the moderator of confirmed COVID-19 case numbers, simple slope analysis indicated the association to also be positively significant in counties with more confirmed COVID-19 cases (*B* = 0.326, *SE* = 0.039, *p* < 0.01), whereas a weaker association in the same direction was found in counties with fewer cases (*B* = 0.166, *SE* = 0.036, *p* = 0.020).

## 4. Discussion

The findings of the present study, conducted during the early stages of the COVID-19 pandemic, support the hypothesis that both forms of others-oriented anxiety (i.e., anxiety about others’ health and anxiety about societal problems) are positively associated with the frequency of physical distancing adoption beyond the influence of self-oriented anxiety concerning the possibility of contracting COVID-19 oneself. Widespread public anxiety has been observed across the globe amid the COVID-19 pandemic. Public health professionals have issued warnings about the negative implications of such public fear for individual wellbeing and societal functioning (e.g., mass panic, the hoarding of essential supplies) [44]. However, the findings reported herein indicate that anxious responses toward the pandemic, especially anxiety concerning others’ wellbeing and societal problems, may be adaptive in raising awareness of the need for preventive measure adoption.

The positive association identified between both forms of others-oriented anxiety and physical distancing adoption further corroborates the growing body of literature postulating this preventive measure as a form of prosocial response to the pandemic, a response motivated by empathetic concern over the wellbeing of others. For instance, prior studies conducted during the COVID-19 pandemic found trait empathy and perceived social responsibilities to be associated with physical distancing adoption [45,46].

More importantly, the person–environment interactionist approach adopted in the current research further demonstrated the magnitude of the positive association between others-oriented anxiety and physical distancing adoption to vary across the residents of diverse communities. In partial support of the study’s hypotheses, that association tended to be weaker among participants living in socially vulnerable communities (i.e., those with a low SES and poor housing conditions) and in communities heavily affected by the pandemic (i.e., those with a high number of confirmed COVID-19 cases).

The present findings highlight the importance of acknowledging and examining the pivotal role of socioeconomic factors in the adoption of preventive measures. For instance, past disaster research has demonstrated that a lack of resources in vulnerable communities can attenuate community members’ incentive to proactively adopt preventive measures, leaving them more reliant on external help, such as government intervention [47]. Research conducted during the ongoing COVID-19 pandemic has similarly found the pandemic’s economic impact on low-income communities to be a key factor in physical distancing adoption [48]. As those living in low-income communities are more likely than those living in high- and middle-income communities to be employed in occupations that do not allow remote working arrangements (e.g., construction workers, those employed in the service industry), they are often pressured to remain in or return to their workplaces to avoid adverse financial repercussions [49], which they may view as the more imminent adverse impact on their lives. Hence, anxiety may play a less influential role for the residents of low (vs. high) -income communities because physical distancing practice may jeopardize their livelihoods.

### 4.1. Practical Implications

This study has several implications for mental health professionals and policymakers. First, in contrast to previous observations made by mental health researchers that high anxiety levels can prevent rational thinking and responses to the COVID-19 pandemic [50,51], our findings indicate that during the early stages of a pandemic, anxiety concerning one’s own self-interest and the wellbeing of others may play a protective role in alerting people to the need to adopt preventive measures, such as physical distancing, particularly in low-risk communities. It is, thus, important that mental health professionals remain mindful of the need to differentiate individuals with pathological levels of anxiety and problematic thoughts or behaviors derived from such anxiety (e.g., suicidal thoughts) from those who merely feel anxious about the threat posed by the pandemic, for which no currently effective solutions have been proposed for curbing the outbreak within the U.S. More resources should be dedicated to helping the former group of individuals, especially those with pre-existing risk factors (e.g., a history of mental health issues), through the provision of online mental health support services.

Second, in the context of the current pandemic, where individual compliance with government-mandated health policies confers collective benefits, but large-scale enforcement is costly and controversial, communication and persuasion may play a pivotal role. Hence, a rapidly growing body of research is investigating how individuals can be encouraged to undertake such preventive measures as physical distancing. These studies have demonstrated that public messages, appealing to either social responsibility or empathy (e.g., the responsibility of every citizen to protect the wellbeing of others) or anxiety and fear about the pandemic (e.g., the serious outcomes of contracting the virus), are effective in promoting physical distancing [13]. Our findings concerning others-oriented anxiety further indicate that the scope of public messages should be expanded to include the interests and wellbeing of close social network members and society as a whole. Public education programs on infectious disease prevention should highlight the possibility that a failure to adopt preventive measures is hazardous not only to one’s own health, but also to that of close social network members and people in the community.

Finally, public education and announcements may not be the most effective means of encouraging physical distancing adoption, especially if such adoption could have serious financial consequences. As our findings indicate that others-oriented anxiety is associated with physical distancing adoption to a lesser extent among residents of high-risk, vulnerable communities, relative to those living in low-risk communities, policymakers should consider utilizing diverse health control strategies to promote such adoption in the former. For instance, scholars have proposed that to promote physical distancing adoption among residents of vulnerable communities who may experience financial hardship amid the pandemic, public health campaigns should provide advice on how to practice physical distancing while still accessing basic income and resources [52], as well as messages fostering self-efficacy [53].

### 4.2. Research Caveats

Although the study reported herein was the first to investigate the role of social-oriented anxiety and community risk factors in physical distancing adoption, it was not without limitations. First, it was conducted during a relatively early stage of the pandemic (18–19 March 2020), when no U.S. state had yet officially announced a stay-at-home order. At the time of study, the participants had only been advised not to travel outdoors, not mandated to stay home. In addition, many industries (e.g., the service industry) were still requiring their employees to show up for work. Hence, data collected during this period are able to capture individual differences in voluntary physical distancing adoption. Recent reports indicate that the implementation of stay-at-home orders has dramatically reduced the amount of time people spend outside their places of residence [54], with such implementation, thus, minimizing individual differences. Although they capture such differences, our findings may not be generalizable to later stages of the COVID-19 pandemic. Previous findings have shown that levels of public anxiety varied considerably across various phases during the SARS outbreak [55], so multi-wave longitudinal design should be adopted in future research to investigate the trajectory of changes in public sentiment, across various waves of the COVID-19 pandemic.

Second, an online survey method was used to collect self-report data. As this method relies primarily on closed-ended items, such as multiple-choice questions, the item format restricts participants’ ability to explain and provide further insight into their behaviors (e.g., why they have chosen not to adopt preventive measures during the COVID-19 pandemic). However, as the study was conducted amid the pandemic, conducting face-to-face interviews would have posed technical difficulties, as several states and counties had already advised citizens not to go outside or attend public events. Conducting an online survey allowed us to recruit a sample of community adults, despite the pandemic. Furthermore, recent studies based on GPS-tracked mobility data recording individuals’ travel history have shown self-report measures of physical distancing adoption to be largely consistent with actual behavior [56], providing some empirical support for the use of such measures in community research.

Third, the present study did not include any measures on digital competence and use of digital devices, both of which played vital roles in health information dissemination when the physical distancing health control measures were in place. Studies have shown that residents from COVID-19-affected regions relied heavily on digital devices for seeking information and advice from their social network members or other Internet users, through online forums and social networking sites [57,58]. It is noteworthy that individuals differ considerably in the level of digital competence, with youngsters born and raised in the digital era being more comfortable and competent in information technology use, while older people faced more problems using digital devices that they were not familiar with [59,60]. More research effort should be expended in studying such cohort differences in digital competence and its possible impact on the knowledge acquisition process, with regard to physical distancing practices observed for meeting the government-mandated restrictions for COVID-19 infection prevention.

## 5. Conclusions

In conclusion, this study provides evidence indicating that anxiety may serve a health-protective role during a pandemic because feelings of anxiety, particularly concerning the wellbeing of others, may motivate the adoption of preventive measures, such as physical distancing. However, the study also unveils further intricacies based on community risk factors. For example, the positive association documented between others-oriented anxiety and physical distancing adoption in this research tends to be stronger among participants living in low-risk communities (i.e., communities characterized by a relatively low number of confirmed cases, high SES, and good housing conditions). During the early stage of a pandemic, when the individual adoption of preventive measures confers collective benefits, but the strict enforcement of such measures can be costly and controversial, public messages and communication can play an important role in promoting this adoption. Hence, knowledge of the underlying factors associated with the adoption of physical distancing constitutes useful information for health professionals and policymakers seeking to promote preventive measures among diverse sectors of U.S. society in an effective manner.

Apart from studying physical distancing adoption as an outcome, it is also important to investigate the aftermath of adopting physical distancing measures, especially on students and young adults. The unprecedented COVID-19 pandemic has created a “new normal” that has immensely changed the daily lives of many people in the affected regions. For instance, in order to curb the rapid transmission of the atypical virus, many countries have required people to observe physical distancing and, thus, employees and students need to work or study from home [61,62]. Studies have identified a myriad of problems encountered during home-based teleworking and distance learning, such as adjustment to new work or learning modes and arrangements, obstacles to task accomplishment and performance, and communication breakdowns [63,64,65,66]. More scholarly and professional attention should, thus, be expended on working with such multiple issues, which emerged after physical distancing adoption, and such effort will offer valuable insights to mitigate pandemic-related anxiety, especially for young people with limited life experiences for coping with the drastic vicissitudes brought by the unprecedented, ongoing pandemic.

## Figures and Tables

**Table 1:** Community risk factors as moderators in the association between two forms of pandemic-specific anxiety and physical distancing adoption.

Step and Variable	Model 1: Anxiety Over Others’ Health		Model 2: Anxiety Over Societal Problems	
Step 1	*R*^2^ = 0.067		*R*^2^ = 0.065	
Age	0.093 ***	0.027	0.093 ***	0.027
Self-oriented anxiety	0.173 ***	0.031	0.173 ***	0.031
Step 2	Δ*R*^2^ = 0.038 ***		Δ*R*^2^ = 0.035 ***	
Age	0.154 ***	0.038	0.154 ***	0.039
Self-oriented anxiety	0.131 ***	0.046	0.113 ***	0.033
Others-oriented anxiety	0.167 ***	0.045	0.171 ***	0.042
COVID-19 case number	0.034	0.055	0.029	0.055
Socioeconomic status (SVI-1)	−0.056	0.068	−0.065	0.069
Household composition (SVI-2)	−0.039	0.063	−0.043	0.065
Minority status (SVI-3)	0.065	0.058	0.055	0.058
Housing type (SVI-4)	−0.101 *	0.055	−0.110 *	0.056
Step 3	Δ*R*^2^ = 0.022 ***		Δ*R*^2^ = 0.024***	
Age	0.144 ***	0.038	0.156 ***	0.039
Self-oriented anxiety	0.112 **	0.027	0.104 *	0.027
Others-oriented anxiety	0.125 ***	0.039	0.155 ***	0.047
COVID-19 case number	0.032	0.052	0.027	0.054
Socioeconomic status (SVI-1)	−0.057	0.068	−0.054	0.068
Household composition (SVI-2)	−0.039	0.064	−0.029	0.064
Minority status (SVI-3)	0.064	0.058	0.049	0.058
Housing type (SVI-4)	−0.099 *	0.055	−0.094 *	0.055
Anxiety × COVID-19 case number	−0.086	0.053	−0.111 *	0.053
Anxiety × Socioeconomic status (SVI-1)	−0.198 **	0.066	−0.186 **	0.064
Anxiety × Household composition (SVI-2)	0.100	0.054	0.091	0.053
Anxiety × Minority status (SVI-3)	0.065	0.054	0.081	0.056
Anxiety × Housing type (SVI-4)	−0.144 *	0.065	−0.156 **	0.064

* *p* < 0.05, ** *p* < 0.01, *** *p* < 0.001.

## Data Availability

The data presented in this study are available on request from the authors. The data are not publicly available due to the adherence to the conditions stated in the research protocol submitted for human research ethical approval.

## References

[1] Kola L., Kohrt B.A., Hanlon C., Naslund J.A., Sikander S., Balaji M., Benjet C., Cheung E.Y.L., Eaton J., Gonsalves P. (2021). COVID-19 mental health impact and responses in low-income and middle-income countries: Reimagining global mental health. Lancet Psychiat..

[2] Adiukwu F., de Filippis R., Orsolini L., Gashi Bytyçi D., Shoib S., Ransing R., Slaih M., Jaguga F., Handuleh J.I.M., Ojeahere M.I. (2022). Scaling up global mental health services during the COVID-19 pandemic and beyond. Psychiatr. Serv..

[3] Chen S., Guo L., Alghaith T., Dong D., Alluhidan M., Hamza M.M., Herbst C.H., Zhang X., Tagtag G.C.A., Zhang Y. (2021). Effective COVID-19 control: A comparative analysis of the stringency and timeliness of government responses in Asia. Int. J. Environ. Res. Public Health.

[4] Peeling R.W., Heymann D.L., Teo Y.-Y., Garcia P.J. (2022). Diagnostics for COVID-19: Moving from pandemic response to control. Lancet.

[5] Long N. (2020). From social distancing to social containment. Med. Anthropol. Theory.

[6] Bowman L., Kwok K.O., Redd R., Yi Y., Ward H., Wei W.I., Atchison C., Wong S.Y.-S. (2021). Comparing public perceptions and preventive behaviors during the early phase of the COVID-19 pandemic in Hong Kong and the United Kingdom: Cross-sectional survey study. J. Med. Internet Res..

[7] Toledo-Fernández A., Betancourt-Ocampo D., González-González A. (2021). Distress, depression, anxiety, and concerns and behaviors related to COVID-19 during the first two months of the pandemic: A longitudinal study in adult Mexicans. Behav. Sci..

[8] Lazarus R.S. (1999). Stress and Emotion: A New Synthesis.

[9] Taha S.A., Matheson K., Anisman H. (2014). H1N1 was not all that scary: Uncertainty and stressor appraisals predict anxiety related to a coming viral threat. Stress Health.

[10] Ibuka Y., Chapman G.B., Meyers L.A., Li M., Galvani A.P. (2010). The dynamics of risk perceptions and precautionary behavior in response to 2009 (H1N1) pandemic influenza. BMC Infect. Dis..

[11] Liao Q., Cowling B.J., Lam W.W.T., Ng D.M.W., Fielding R. (2014). Anxiety, worry and cognitive risk estimate in relation to protective behaviors during the 2009 influenza A/H1N1 pandemic in Hong Kong: Ten cross-sectional surveys. BMC Infect. Dis..

[12] Harper C.A., Satchell L.P., Fido D., Latzman R.D. (2021). Functional fear predicts public health compliance in the COVID-19 pandemic. Int. J. Ment. Health Addict..

[13] Heffner J., Vives M.-L., FeldmanHall O. (2021). Emotional responses to prosocial messages increase willingness to self-isolate during the COVID-19 pandemic. Pers. Individ. Differ..

[14] Huarcaya-Victoria J., Villarreal-Zegarra D., Podestà A., Luna-Cuadros M.A. (2022). Psychometric properties of a Spanish version of the fear of COVID-19 scale in general population of Lima, Peru. Int. J. Ment. Health Addict..

[15] Perz C.A., Lang B.A., Harrington R. (2022). Validation of the Fear of COVID-19 Scale in a US College Sample. Int. J. Ment. Health Addict..

[16] Yesudhas D., Srivastava A., Gromiha M.M. (2021). COVID-19 outbreak: History, mechanism, transmission, structural studies and therapeutics. Infection.

[17] Immordino G., Jappelli T., Oliviero T., Zazzaro A. (2022). Fear of COVID-19 contagion and consumption: Evidence from a survey of Italian households. Health Econ..

[18] Nabe-Nielsen K., Nilsson C.J., Juul-Madsen M., Bredal C., Hansen L.O.P., Hansen Å.M. (2021). COVID-19 risk management at the workplace, fear of infection and fear of transmission of infection among frontline employees. Occup. Environ. Med..

[19] Freidin E., Acera Martini L., Senci C.M., Duarte C., Carballo F. (2022). Field observations and survey evidence to assess predictors of mask wearing across different outdoor activities in an Argentine city during the COVID-19 pandemic. Appl. Psychol..

[20] Caycho-Rodríguez T., Valencia P.D., Vilca L.W., Carbajal-León C., Vivanco-Vidal A., Saroli-Araníbar D., Reyes-Bossio M., White M., Rojas-Jara C., Polanco-Carrasco R. (2022). Prevalence and predictors of intention to be vaccinated against COVID-19 in thirteen Latin American and Caribbean countries. Trends Psychol..

[21] Jung H., Albarracín D. (2021). Concerns for others increase the likelihood of vaccination against influenza and COVID-19 more in sparsely rather than densely populated areas. Proc. Natl. Acad. Sci. USA.

[22] Xu P., Cheng J. (2021). Individual differences in social distancing and mask-wearing in the pandemic of COVID-19: The role of need for cognition, self-control and risk attitude. Pers. Individ. Differ..

[23] Cheng C., Wang H.-y., Chan L. (2021). Multiple forms of mass anxiety in Coronavirus Disease-2019 pandemic. J. Affect. Disord..

[24] Cheng C., Wang H.-Y., Ebrahimi O.V. (2021). Adjustment to a “new normal:” Coping flexibility and mental health issues during the COVID-19 pandemic. Front. Psychiatry.

[25] Vezzali L., Cadamuro A., Versari A., Giovannini D., Trifiletti E. (2015). Feeling like a group after a natural disaster: Common ingroup identity and relations with outgroup victims among majority and minority young children. Br. J. Soc. Psychol..

[26] Johns Hopkins Coronavirus Resource Center Coronavirus COVID-19 Global Cases. https://coronavirus.jhu.edu/map.html.

[27] Flanagan B.E., Gregory E.W., Hallisey E.J., Heitgerd J.L., Lewis B. (2011). A social vulnerability index for disaster management. J. Homel. Secur. Emerg. Manag..

[28] Placzek H., Madoff L. (2014). Effect of race/ethnicity and socioeconomic status on pandemic H1N1-related outcomes in Massachusetts. Am. J. Public Health.

[29] Crouse Quinn S. (2008). Crisis and emergency risk communication in a pandemic: A model for building capacity and resilience of minority communities. Health Promot. Pract..

[30] Mischel W. (2004). Toward an integrative science of the person. Annu. Rev. Psychol..

[31] Endler N.S., Parker J.D. (1992). Interactionism revisited: Reflections on the continuing crisis in the personality area. Eur. J. Pers..

[32] Flett G.L., Endler N.S., Besser A. (2009). Separation anxiety, perceived controllability, and homesickness. J. Appl. Soc. Psychol..

[33] Sorić I. (1999). Anxiety and coping in the context of a school examination. Soc. Behav. Pers..

[34] Blanchard C.M., Rodgers W.M., Bell G., Wilson P.M., Gesell J. (2002). An empirical test of the interaction model of anxiety in an acute exercise setting. Pers. Individ. Differ..

[35] Gaudron J.-P., Vignoli E. (2002). Assessing computer anxiety with the interaction model of anxiety: Development and validation of the computer anxiety trait subscale. Comput. Hum. Behav..

[36] Gray L., MacDonald C., Mackie B., Paton D., Johnston D., Baker M.G. (2012). Community responses to communication campaigns for influenza A (H1N1): A focus group study. BMC Public Health.

[37] Santibanez T.A., Singleton J.A., Santibanez S.S., Wortley P., Bell B.P. (2013). Socio-demographic differences in opinions about 2009 pandemic influenza A (H1N1) and seasonal influenza vaccination and disease among adults during the 2009–2010 influenza season. Influenza Other Respir. Viruses.

[38] Cheng C., Wang H.-y., Chau C.-l. (2021). Mental health issues and health disparities amid COVID-19 outbreak in China: Comparison of residents inside and outside the epicenter. Psychiatry Res..

[39] Bults M., Beaujean D.J.M.A., Wijkmans C.J., Timen A., Richardus J.H., Voeten H.A.C.M. (2012). Why did patients with cardiovascular disease in the Netherlands accept Q fe.ever vaccination?. Vaccine.

[40] Olofsson A. (2011). Organizational crisis preparedness in heterogeneous societies: The OCPh model. J. Conting. Crisis Manag..

[41] Cheng C., Cheung M.W.L. (2005). Psychological responses to outbreak of severe acute respiratory syndrome: A prospective, multiple time-point study. J. Pers..

[42] Kounadi O., Lampoltshammer T.J., Leitner M., Heistracher T. (2013). Accuracy and privacy aspects in free online reverse geocoding services. Cartogr. Geogr. Inf. Sci..

[43] R Core Team (2020). A Language and Environment for Statistical Computing.

[44] Micalizzi L., Zambrotta N.S., Bernstein M.H. (2021). Stockpiling in the time of COVID-19. Br. J. Health Psychol..

[45] Galang C.M., Johnson D., Obhi S.S. (2021). Exploring the relationship between empathy, self-construal style, and self-reported social distancing tendencies during the COVID-19 pandemic. Front. Psychol..

[46] Hills S., Eraso Y. (2021). Factors associated with non-adherence to social distancing rules during the COVID-19 pandemic: A logistic regression analysis. BMC Public Health.

[47] Shaw D., Scully J., Hart T. (2014). The paradox of social resilience: How cognitive strategies and coping mechanisms attenuate and accentuate resilience. Glob. Environ. Change.

[48] Wright A.L., Sonin K., Driscoll J., Wilson J. (2020). Poverty and economic dislocation reduce compliance with COVID-19 shelter-in-place protocols. J. Econ. Behav. Organ..

[49] Kavanagh N.M., Goel R.R., Venkataramani A.S. (2021). County-level socioeconomic and political predictors of distancing for COVID-19. Am. J. Prev. Med..

[50] Ahorsu D.K., Lin C.-Y., Imani V., Saffari M., Griffiths M.D., Pakpour A.H. (2020). The Fear of COVID-19 scale: Development and initial validation. Int. J. Ment. Health Addict..

[51] Ying W., Cheng C. (2021). Public emotional and coping responses to the COVID-19 infodemic: A review and recommendations. Front. Psychiatry.

[52] Bonell C., Michie S., Reicher S., West R., Bear L., Yardley L., Curtis V., Amlôt R., Rubin G.J. (2020). Harnessing behavioural science in public health campaigns to maintain ‘social distancing’ in response to the COVID-19 pandemic: Key principles. J. Epidemiol. Community Health.

[53] Vaughan E., Tinker T. (2009). Effective health risk communication about pandemic influenza for vulnerable populations. Am. J. Public Health.

[54] Wellenius G.A., Vispute S., Espinosa V., Fabrikant A., Tsai T.C., Hennessy J., Dai A., Williams B., Gadepalli K., Boulanger A. (2021). Impacts of social distancing policies on mobility and COVID-19 case growth in the US. Nat. Commun..

[55] Cheng C., Tang C.S. (2004). The psychology behind the masks: Psychological responses to the severe acute respiratory syndrome outbreak in different regions. Asian J. Soc. Psychol..

[56] Gollwitzer A., McLoughlin K., Martel C., Marshall J., Höhs J.M., Bargh J.A. (2021). Linking self-reported social distancing to real-world behavior during the COVID-19 pandemic. Soc. Psychol. Pers. Sci..

[57] Cheng C., Ebrahimi O.V., Lau Y. (2020). Maladaptive coping with the infodemic and sleep disturbance in the COVID-19 pandemic. J. Sleep Res..

[58] Rosen A.O., Holmes A.L., Balluerka N., Hidalgo M.D., Gorostiaga A., Gómez-Benito J., Huedo-Medina T.B. (2022). Is social media a new type of social support? social media use in Spain during the COVID-19 pandemic: A mixed methods study. Int. J. Environ. Res. Public Health.

[59] Repique R.J.R. (2013). Digital natives, digital immigrants: Dichotomy or diversity in psychiatric nursing?. J. Am. Psychiatr. Nurses Assoc..

[60] Teo T. (2013). An initial development and validation of a Digital Natives Assessment Scale (DNAS). Comput. Educ..

[61] Aretio L.G. (2021). COVID-19 and digital distance education: Pre-confinement, confinement and post-confinement. Ried-Rev. Iberoam. De Educ. Distancia.

[62] Escudero-Castillo I., Mato-Díaz F., Rodriguez-Alvarez A. (2021). Furloughs, teleworking and other work situations during the COVID-19 lockdown: Impact on mental well-being. Int. J. Environ. Res. Public Health.

[63] García-Morales V.J., Garrido-Moreno A., Martín-Rojas R. (2021). The transformation of higher education after the COVID disruption: Emerging challenges in an online learning scenario. Front. Psychol..

[64] Azzi D.V., Melo J., Neto A.D.A.C., Castelo P.M., Andrade E.F., Pereira L.J. (2021). Quality of life, physical activity and burnout syndrome during online learning period in Brazilian university students during the COVID-19 pandemic: A cluster analysis. Psychol Health Med..

[65] Pulido-Martos M., Cortés-Denia D., Lopez-Zafra E. (2021). Teleworking in times of COVID-19: Effects on the acquisition of personal resources. Front. Psychol..

[66] Buomprisco G., Ricci S., Perri R., De Sio S. (2021). Health and telework: New challenges after COVID-19 pandemic. Eur. J. Environ. Public Health.

